# A scoping review on associations between paratuberculosis and productivity in cattle

**DOI:** 10.3389/fvets.2024.1352623

**Published:** 2024-05-02

**Authors:** Silja Griss, Tanja Knific, Anne Buzzell, Luís Pedro Carmo, Gertraud Schüpbach-Regula, Mireille Meylan, Matjaž Ocepek, Beat Thomann

**Affiliations:** ^1^Vetsuisse Faculty, Veterinary Public Health Institute, University of Bern, Bern, Switzerland; ^2^Graduate School for Cellular and Biomedical Sciences, University of Bern, Bern, Switzerland; ^3^Veterinary Faculty, Institute of Food Safety, Feed and Environment, University of Ljubljana, Ljubljana, Slovenia; ^4^Norwegian Veterinary Institute, Ås, Norway; ^5^Clinic for Ruminants, Vetsuisse Faculty, University of Bern, Bern, Switzerland; ^6^Veterinary Faculty, Institute of Microbiology and Parasitology, University of Ljubljana, Ljubljana, Slovenia

**Keywords:** *Mycobacterium avium* subsp. paratuberculosis, Johne’s disease, production effects, disease effects, disease impact, dairy cow, beef cattle

## Abstract

Paratuberculosis (PTB), or Johne’s disease, is a disease with worldwide distribution caused by *Mycobacterium avium* subsp*. paratuberculosis* (MAP) that leads to chronic enteritis, primarily in ruminants. Even subclinical infection significantly reduces the animals’ performance, and consequences of the disease lead to high economic losses for the cattle industry. To estimate the economic burden of bovine PTB and to evaluate the benefits of a potential control program, accurate estimates of the production effects associated with the disease are required. Therefore, the aim of this scoping review was to provide a comprehensive overview of associations between MAP infection and production parameters in cattle. The studies were collected from three electronic databases. Of the total 1,605 identified studies, 1,432 did not meet the set criteria in the title and abstract screening and a further 106 were excluded during full-text review. Finally, data on 34 different production parameters were extracted from 67 publications. Results show that the magnitude of reported performance losses varies depending on several factors, such as the type of diagnostic test applied, disease status or number of lactations. Studies reported a reduction in milk yield, changes in milk quality (e.g., higher somatic cell count, lower amount of produced milk fat and protein), reduced fertility (e.g., prolonged calving interval and service period, higher abortion rate and calving difficulties), reduced weaning weight, slaughter weight and slaughter value, or a higher risk for mastitis. Results from the studies included in our review show a median decrease of milk yield per infected cow of −452 kg/lactation for raw and −405 kg/lactation for modeled data. Similarly, the amount of produced milk protein fell by a median of −14.41 kg/lactation for modeled data and the amount of produced milk fat by a median of −13.13 kg/lactation. The reviewed studies revealed a prolonged calving interval by around 30 days and a 1.5 to 3 times higher likeliness of culling per lactation in PTB positive animals. Results from this scoping review provide evidence-based inputs for the development of economic models aiming at the estimation of the costs and benefits associated with different disease control scenarios for PTB.

## 1 Introduction

*Mycobacterium avium* subsp. *paratuberculosis* (MAP) occurs worldwide and is the etiological pathogen of the disease known as paratuberculosis (PTB) or Johne’s disease (JD) (1). It causes chronic enteritis (2), primarily in ruminants, but also occurs in other animals (e.g., rabbits, foxes, weasels) (1). Calves can become infected mainly via the fecal-oral route or through colostrum and milk from infected animals, but other transmission routes, including *in utero*, are also described (3). Due to the long incubation period of two to seven years (4), many cows in a herd infected with MAP are asymptomatic, while only few show the typical clinical signs of PTB, diarrhea and weight loss (5). However, even subclinical infection significantly reduces the performance of the animals and consequences of the disease lead to high direct and indirect economic losses for the cattle industry (6). Reasons for the economic losses are diverse and include reduced milk yield and quality, decreased slaughter weight and value, decreased fertility and increased susceptibility to other chronic diseases, among others (7). These losses in animal productivity are one of the key drivers for efforts to control the disease in many countries of Europe (8). Overall, an annual loss of 198 million US$ is estimated for the U.S. dairy industry due to PTB (7). Annual losses per infected cow in the USA are estimated between 21 US$ and 78 US$ (8). Tiwari et al. (9) estimated a mean annual loss of 2,992 Can$ (2,196 US$) per infected herd or 49 Can$ (36 US$) per infected cow. Estimations from Europe show losses of 234 € (251 US$) per cow in France and 27 GBP (33 US$) in the UK (8). Rieger et al. (10) performed a meta-analysis to estimate economic losses caused by reduced milk yield and reproductive performance associated with bovine PTB in Switzerland. They calculated a loss of 358.83 CHF per infected cow. Few studies also suggest that MAP may potentially pose a risk to human health as it has been isolated from the intestines of patients with Crohn’s disease (11). Increasing public health concerns (12) might lead to trade restrictions for dairy products in the future and thus to further indirect losses (8).

To estimate the economic burden of bovine PTB and to evaluate the benefits of a potential control program, accurate estimates of the production effects associated with the disease are required (13). Several studies examined the economic consequences of PTB infection on a specific production parameter, e.g., milk yield, slaughter weight or reproduction indicators. However, the estimated effect size varies considerably due to various factors, such as test method, production system, herd size, herd management, case definition, geographical area and/or disease prevalence (14). Currently, a comprehensive systematic review with meta-analysis exists only for the production parameter milk yield, in which a reduction of milk yield of −1.87 kg per day or −5.9% of yield for fecal culture or PCR positive cows was reported (13). Two further reviews exist, in which various production parameters and the economic effect of PTB were evaluated (6, 14). However, these two reviews are rather narrative, and do not represent comprehensive reports on the existing literature. To the best of our knowledge, no structured review following a systematic approach has been conducted on the association between a PTB test positive cow or herd status and different production parameters. The aim of this scoping review was to identify factors of relevance for economic impact estimation, to define the extent of the losses for each production parameter, and to provide evidence-based inputs for the development of economic models for bovine PTB impact estimations.

## 2 Materials and methods

We performed a scoping review in accordance with the Preferred Reporting Items Extension for Scoping Reviews (PRISMA-ScR) (15), using a systematic search strategy to identify literature on a topic, extracted data from relevant papers and synthesized the results (16). The methodology of scoping review rather than a systematic review was chosen because the study question was relatively broad. The protocol of this review was registered at SYREAF (Systematic Reviews for Animals and Food) (17) or can be found on the repository BORIS (18).

### 2.1 Eligibility criteria

Primary research, investigating on the relationship between bovine PTB/JD and production parameters in cattle (e.g., milk yield, milk quality, fertility parameters, slaughter weight or slaughter value) were included in our review. The reported association with productivity had to be quantified to be included in the review. The association between MAP and productivity in other animals (e.g., goats, sheep) or the economic consequences of control programs were not considered. All publications that met these criteria, were available in full-text and written in English, French, German, or Slovenian were eligible for our scoping review.

### 2.2 Literature search

The search string was developed iteratively on the basis of the PICo elements (Problem, Interest, Context). Defining search terms in an iterative process rather than a strictly pre-defined list of search terms was more efficient to achieve inclusion of all relevant scientific literature. The specific PICo elements were (1) Problem: Paratuberculosis, (2) Interest: Economic impact, (3) Context: Cattle. For each PICo element different key words were identified by experts in our review team and subject heading search was used. The electronic databases PubMed (19), CAB Direct (20) and Web of Science (21) were searched for literature in August 2022, using the final search string presented in Table 1. In addition, the reference lists of relevant reviews were screened to identify and include other potentially useful studies.

**Table 1:** Search string applied in the scoping review to identify literature on the associations between paratuberculosis and productivity in cattle.

Search string	
#1 Problem	Paratuberculosis OR “*Mycobacterium avium* subspecies paratuberculosis” OR “Johne’s disease”
	AND
#2 Interest	“Milk production” OR “Milk quality” OR “Milk yield” OR “Somatic cell count” OR “Milk fat” OR “Milk protein” OR “Slaughter weight” OR “Weaning weight” OR “Culling rate” OR “Mortality” OR “replacement” OR “infertility” OR “genetic value” OR “pregnancy rate” OR “Abortion” OR “Non-return rate” OR “mastitis” OR “economic*” OR “production effect*” OR “production loss*” OR cull OR “conception rate” OR cost OR costs OR emaciation
	AND
#3 Context	Cow OR calves OR dam OR herd OR herds OR farm OR farms OR ruminant OR cattle OR bovine OR dairy OR beef

### 2.3 Literature screening

To remove duplicate results and facilitate the screening process among different reviewers, the software Covidence was used (22). For preliminary screening, each study was independently rated by two of three reviewers (SG, TK, AB) based on the titles and abstracts to determine whether it met the eligibility criteria mentioned above. A calibration exercise was performed using the first 10% (*n* = 160) of the identified publications, to ensure mutual understanding of the eligibility criteria and capture of relevant literature. If two reviewers agreed that a specific paper met the eligibility criteria, the paper was included in the full-text screening step.

Two reviewers (SG, TK) independently reviewed the full-text of each paper and reexamined the eligibility criteria, with particular emphasis on whether the association of MAP infection and changes in production parameters was quantified. This review process was again tested with the first 10% (*n* = 17) of items and modified as needed to ensure consistency. In cases of disagreement during the title and abstract screening or during the full-text screening, a third and a fourth reviewer (BT, MO) were involved.

### 2.4 Data extraction

A data charting form was developed to determine which variables to extract and discussed within the review team (Table 2). The form was updated in an iterative process based on discussion between the reviewers. Preliminary data extraction was completed using Covidence’s data extraction tool (extraction 2.0). Results were then exported to MS Excel, where additional data extraction was completed. Two authors (SG, TK) entered data of interest (e.g., study characteristics, study population) for each individual article into the spreadsheet. The extraction template was tested on five papers and adjusted as needed. In the event of disagreements in the data extraction process, a third and a fourth reviewer (BT, MO) were consulted.

**Table 2:** Data charting form used to extract information from the papers to answer the scoping review’s question on associations between paratuberculosis and productivity in cattle.

Variable	Description of items
Study characteristics	Study ID, title, author, year of publication, DOI/PMID, country of the study, study design, start and end year of study
Study population	Farm type, breed, age category included in study, number of herds, number of positive herds, number of negative herds, total number of animals, number of positive animals, number of negative animals, definition of positive animals or herd, herd size, within-herd prevalence, between-herd prevalence, animal-level prevalence
Interest data	Production parameters, diagnostic test, unit (e.g. kg/day, days/lactation, etc.), level (cow, farm), parity, definition of PTB-status group 1 (always more positive than group 2), value of production parameter group 1, definition of PTB-status group 2 (always less positive than group 1), value of production parameter group 2, difference of production parameter between group 1 and group 2, statistical model type, variables of model, *p*-value

### 2.5 Synthesis of results

For studies in which associations between PTB and changes in productivity in cattle were not reported directly, the mean difference was calculated by subtracting the value of the healthy animals from that of diseased animals. Similarly, if only the value of MAP-negative animals and the mean difference of positive animals were given, the value of the performance of the positive animals was calculated by adding the value of the negative animals and the mean difference. In order to be able to better compare the individual production parameters, reported values were converted to the most common unit whenever possible. Pounds (lb) were converted to kilograms (kg) (1 pound = 0.45 kg). Milk yield given in litres (L) was multiplied by 1.02 to obtain kg (10). Milk yield, amount of produced milk fat and milk protein expressed in kg/day were converted to kg/lactation by multiplying it by 305, as this is the standardized lactation length (23). If fat or protein concentration was given as a percentage, this number was multiplied by the average milk yield in kg in the corresponding animal group (positive or negative) to obtain the fat or protein mass in kg (10). Somatic cell count (SCC) was converted to cells/ml. Values given in linear score (LS) were converted with the following formula: SCC = 100 × 2^(LS – 3)^, where SCC is cells μ/L (24).

Descriptive statistical analyses were performed in STATA (25).

Reported empiric raw values were separated from modeled estimates (derived from statistical analyses and corrected for covariates) for the analyses. When the effects of PTB on productivity in cattle for positive herds/animals based on multiple diagnostic tests results (e.g., fecal culture, PCR, milk or serum ELISA) were reported in a study, the effect size for fecal culture was taken into account for estimating the median effect size of a single production parameter over all studies.

## 3 Results

**Figure 1:**
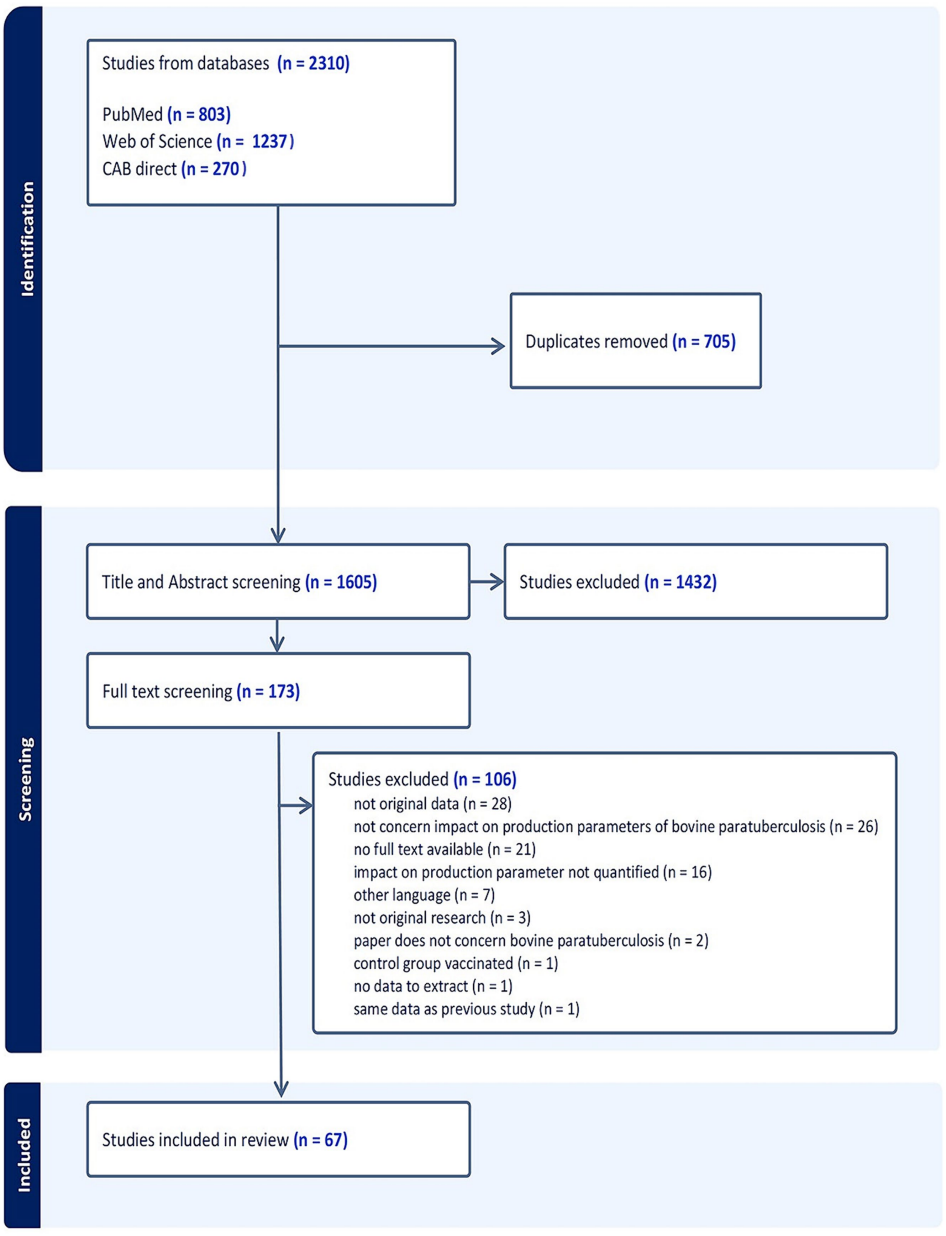
PRISMA flow diagram showing (22) the selection process of the studies included in the scoping review on associations between paratuberculosis and productivity in cattle.

The initial literature search identified a total of 2,310 studies. After deduplication, 1,605 studies were included in title and abstract screening, of which173 were included for full-text review (Figure 1). Of these, 67 papers met the inclusion criteria and were included in the scoping review. No additional papers were found in the reference lists of relevant reviews.

**Figure 2:**
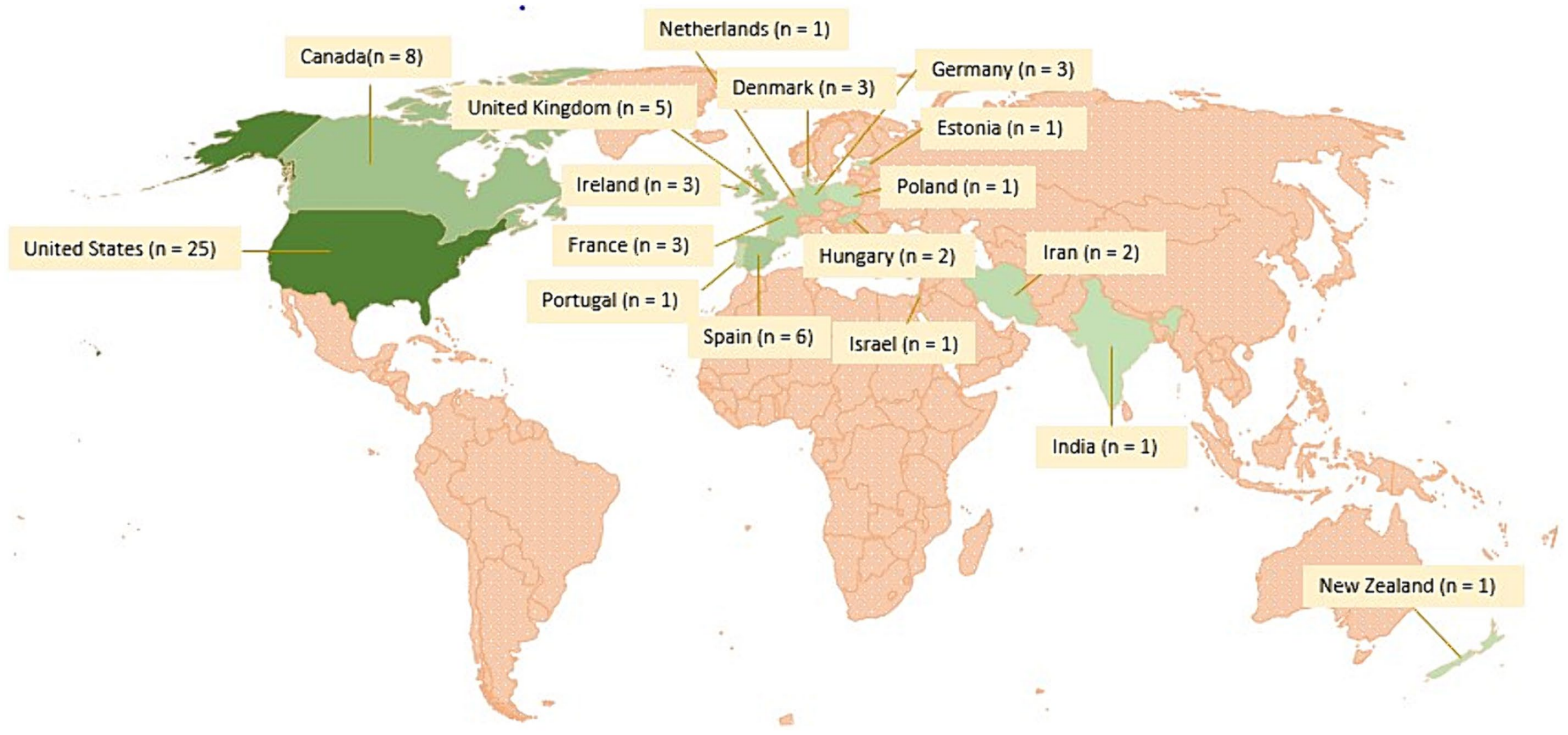
Geographical distribution and number of studies per country included in the scoping review on associations between paratuberculosis and productivity in cattle. The darker the green, the more studies were included from the respective countries. No studies were included from the countries marked in red.

**Figure 3:**
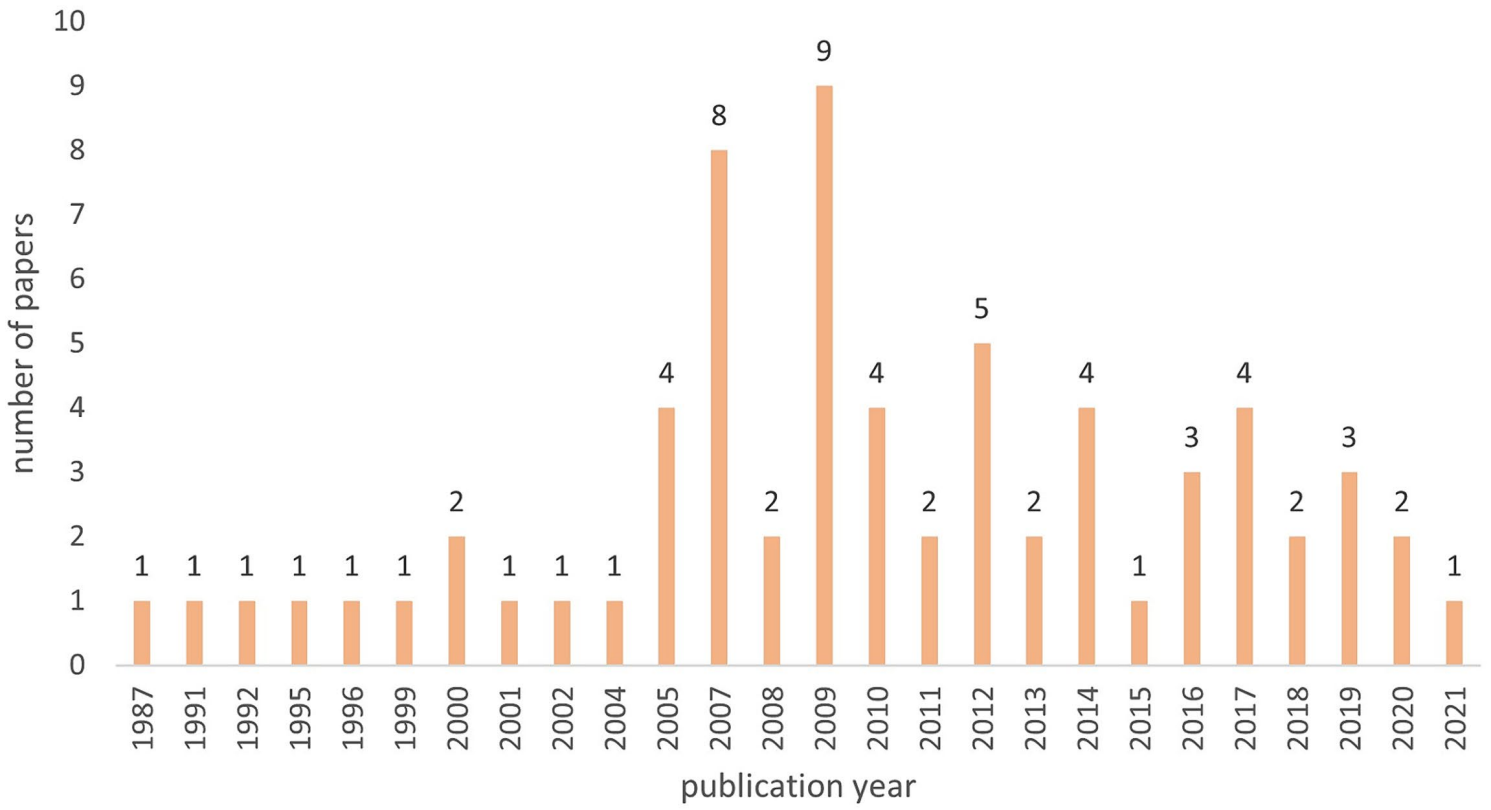
Number of publications per year included in the scoping review on associations between paratuberculosis and productivity in cattle (*n* = 67).

The 67 studies originated from 17 different countries, mostly from North America and Europe (Figure 2). No study from Central and South America or Africa met our inclusion criteria. The articles were published between 1987 and 2021, however over a third (*n* = 27) was published between 2005 and 2010 (Figure 3).

Out of the 67 included studies, 31 were classified as cohort-studies, 28 as cross-sectional and eight as case–control studies. Five studies were conducted on beef farms, while the remaining 62 studies concerned dairy cattle. Only 32 studies indicated the main breed of the herds under study, of which only two were Jersey herds, while all others consisted mainly of Holsteins.

Overall, the associations between MAP infection and 34 different production parameters were described in the 67 articles (Table 3). The most studied parameters were milk yield, other milk parameters (somatic cell count, amount of produced milk fat and protein), culling rate, calving interval and service period. In the following, the most frequently reported production parameters are described in detail.

**Table 3:** Frequency of analysis of the associations between bovine paratuberculosis and a production parameter across all 67 papers of the scoping review.

Number of publications	Production parameter
45	Milk yield
20	Somatic cell count
16	Milk fat, milk protein
14	Culling rate
11	Calving interval
8	Service period
7	Days in milk
6	Longevity, occurrence of mastitis, number of services per conception
4	Calving to first service interval, pregnancy rate
3	Carcass weight, occurrence of lameness, occurrence of pneumonia, non-return rate, laughter value, weaning weight
2	Conception rate, early fetal loss rate, abortion rate, stillbirth rate, occurrence of displaced abomasum, occurrence of ketosis, occurrence of metritis, occurrence of milk fever, occurrence of retained placenta, milk lactose, total milk solids
1	Calving difficulties, calving rate, carcass quality, mortality rate

### 3.1 Milk yield

Associations between PTB status and milk yield were investigated in a total of 45 publications. A significant difference between positive and negative animals or herds was found in 34 of these papers, either on a dichotomous level (Table 4), on various levels of MAP positivity (Table 5) and/or depending on parity (Supplementary Table S1). Five papers where a significant difference in milk yield was found are not listed in any of the tables, because one indicated the milk yield in total milk solids (56), three had a case definition differed from positive vs. negative (clinical vs. non-clinical, before JD vs. after outbreak) (57–59) and one did not specify what type of ELISA test was used (60). A significant decrease in milk yield for positive animals was observed in most studies, but a significant increase in milk yield, was also reported in few publications, depending on the stage of infection (30, 31, 50, 61). In six publications, no significant difference was observed (33, 46, 47, 62–64) and in five publications it was not stated whether the observed difference was significant (34, 44, 49, 65, 66).

**Table 4:** Data from studies (*n* = 26) included in our scoping review, in which associations between bovine paratuberculosis and changes in milk production were investigated dichotomously (positive vs. negative).

Test	Significance	Method	Country	Number of animals	Number of cases	Milk quantification	Milk yield positive animals (kg/305-d lactation)	Milk yield negative animals (kg/305-d lactation)	Mean effect (kg/305-d lactation)	Reference
Serum ELISA	Yes	Modeled	USA	5,926	1,470	kg-FC/test day	10,254.1^b^	11,007.45^b^	−753.35^b^	(26)
			USA	4,375	295	kg/lactation	-	-	−258.6	(27)
			Portugal	22,881	684	kg/5 lactations	-	-	−256.96^b^	(28)
			USA	1,653	147	kg/305 ME	9,145	9,521	−376	(29)
			USA	1,553	200	kg/day	13,185.15^a,b^	13,444.4^b^	−259.25^b^	(30)
			USA	1,653	147	kg/305 ME	9,239.00	9,571.00	−332	(29)
			Poland	454	26	kg/day	9,644.10^b^	9,421.45 ^b^	222.65^b^	(31)
			Poland	424	48	kg/day	9,445.85^b^	9,598.35 ^b^	−183^b^	(31)
	No/not stated	Modeled	Canada	689	130	kg/305-d lactation	7,967.00^a^	8,140	−173	(32)
			USA	2,053	-	kg/day	5,489.00	5,894.00	−405^a^	(33)
			Israel	4,694	-	kg/305-d lactation	-	-	−300	(34)
		Raw	France	15,490	1,139	kg/test day	7,228.5^b^	8,387.5^b^	−1,159 ^a,b^	(35)
Fecal culture	Yes		USA	5,926	1,470	kg-FC/test day	10,348.65^b^	11,007.45^b^	−658.8^b^	(26)
			Germany	4,627	1,382	kg/test day	8,448.5	8,845	−396.5^a^	(36)
			USA	4,375	115	kg/lactation	-	-	−618.7	(27)
		Modeled	Canada	689	72	kg/305-d lactation	7,592^a^	8,140	−548	(32)
			Germany	9,367	1,136	kg/305-d lactation	9,012	9,237	−225	(37)
			USA	655	21	kg/305-d lactation	9,630	10,985	−1,355	(38)
			USA	1,553	200	kg/day	13,121.1^a,b^	13,508.45^b^	−387.35^b^	(30)
			USA	224	84	lbs/305 ME	6,417.88^b^	7,903.39 ^b^	−1,485.52^b^	(39)
		Raw	Germany	279	93	kg/lactation	8,719	9,749	−1,030	(40)
	No/not stated	Raw	France	15,490	143^a^	kg/test-day	7,716.50^b^	8,387.50 ^b^	−671^a,b^	(35)
			USA	289	112	lbs/305 ME	9,166.65^b^	9,372^b^	−452^a,b^	(41)
Milk ELISA	Yes		Canada	689	77	kg/305-d lactation	7,683^a^	8,140	−457	(32)
		Modeled	Hungary	4,341	165	kg/305-d lactation	9,974^b^	11,004 ^b^	−1,030 ^b^	(42)
			Canada	4,389	171	kg/test day	-	-	−1,037	(43)
	No/not stated	Raw	UK	500	48	kg/day	10,156	10,704	−548^a^	(44)
PCR (milk, serum, fecal)	Yes	Modeled	Iran	252	8	kg/305-d lactation	6,263.14	7,272.38 ^a^	−1,009.24	(45)
		Raw	Iran	252	8	kg/305-d lactation	7,392.8	7,641	−248	(45)
	No/not stated	raw	Hungary	30	20	l/day	14,777.25^b^	16,146.09^b^	−1,368.84 ^a,b^	(46)
			France	15,490	1,139	kg/test-day	8,113^b^	8,387.5 ^b^	−274.5^a,b^	(35)
Serum ELISA & fecal culture	Yes	Modeled	USA	4,375	345	kg/lactation	-	-	−303.9	(27)
	No/not stated		USA	5,926	1,470	kg-FC/test day	-	-	−1,433.5^b^	(26)
			USA	533	116	kg/305 ME	-	-	1,412.42	(47)
			Ireland	74	37	kg/lactation	-	-	84.49	(48)
			USA	51,597^a^	4,292^a^	kg/305-d lactation	8,809	9,799	−990	(49)

^a^Calculated values based on given values (difference of healthy animals from that of the diseased animals). ^b^Unit converted to kg/305-day lactation. FC, fat corrected milk. 305 ME = 305-day mature equivalent.

**Table 5:** Data from studies (*n* = 7) in which associations between bovine paratuberculosis and changes in milk production were investigated on multiple levels of positivity included in our scoping review on the associations between paratuberculosis and productivity in cattle.

Test	Case definition	Country	Milk quantification	Mean effect (kg/305-d lactation)	*p*-value	Reference
Fecal culture	Latent	USA	kg/day	701.5^b^	<0.001	(50)
		USA	kg/day	457.5 ^b^	<0.001	(30)
	Low/light shedding	USA	kg/305-d lactation	−537	0.15	(51)
		USA	kg/day	61^b^	0.709	(50)
		USA	kg/day	−417.85^b^	<0.001	(30)
	Moderate shedders	USA	kg/305-d lactation	−1,403	<0.01	(51)
	Heavy/high shedding	USA	kg/305-d lactation	−1,534	<0.01	(51)
		USA	kg/day	−1,128.5 ^b^	0.056	(50)
		USA	kg/day	−1,207.8 ^b^	<0.01	(30)
Serum ELISA	Suspect/inconclusive	USA	lbs/305 ME	−204.57^a,b^	>0.05	(52)
		USA	kg/day of life	39.65	0.719	(53)
		USA	lbs/305 ME	−266.71^b^	>0.05	(54)
	Low positive	USA	lbs/305 ME	−715.77 ^a,b^	-	(52)
		USA	kg/day of life	−6.1^b^	0.982	(53)
	Positive	USA	lbs/305 ME	−606.91^a,b^	<0.05	(52)
		USA	lbs/305 ME	−399.16^a,b^	<0.05	(54)
		USA	kg/day of life	−286.7^b^	0.101	(53)
	Strong positive	USA	lbs/305 ME	−1,136	<0.05	(55)
		USA	lbs/305 ME	−1,364.41^a,b^	<0.05	(54)
		USA	kg/day of life	−573.4^b^	<0.0001	(53)

^a^Calculated values based on given values (difference of healthy animals from that of the diseased animals). ^b^Unit converted to kg/305-day lactation.

Differences in milk yield between MAP positive and negative animals at a dichotomous level ranged from −1,368.84 kg/lactation to 222.65 kg/lactation with a median of −452 kg/lactation for raw data and a median of −405 kg/lactation for modeled data (Table 6).

**Table 6:** Minimum, maximum, and median of dichotomous milk yield mean difference (kg/305-day lactation) in the studies where the associations between bovine paratuberculosis and changes in milk yield included in our scoping review were investigated.

Test	Number of publications	Minimum	Maximum	Median	95% confidence interval
Modeled data (all tests)	17	−1,485.52	1,412.42	−405.00	−986.64; −300.77
Raw data (all tests)*	9	−1,368.84	222.65	−452.00	−1,002.08; −188.06
Serum ELISA (modeled data)	8	−753.35	−173.00	−279.63	−518.21; −229.67
Fecal culture (modeled data)	8	−1,485.52	−225.00	−583.35	−1,397.42; −334.59

*If in a study data with multiple tests were presented, data of fecal culture were used due to their high specificity.

**Figure 4:**
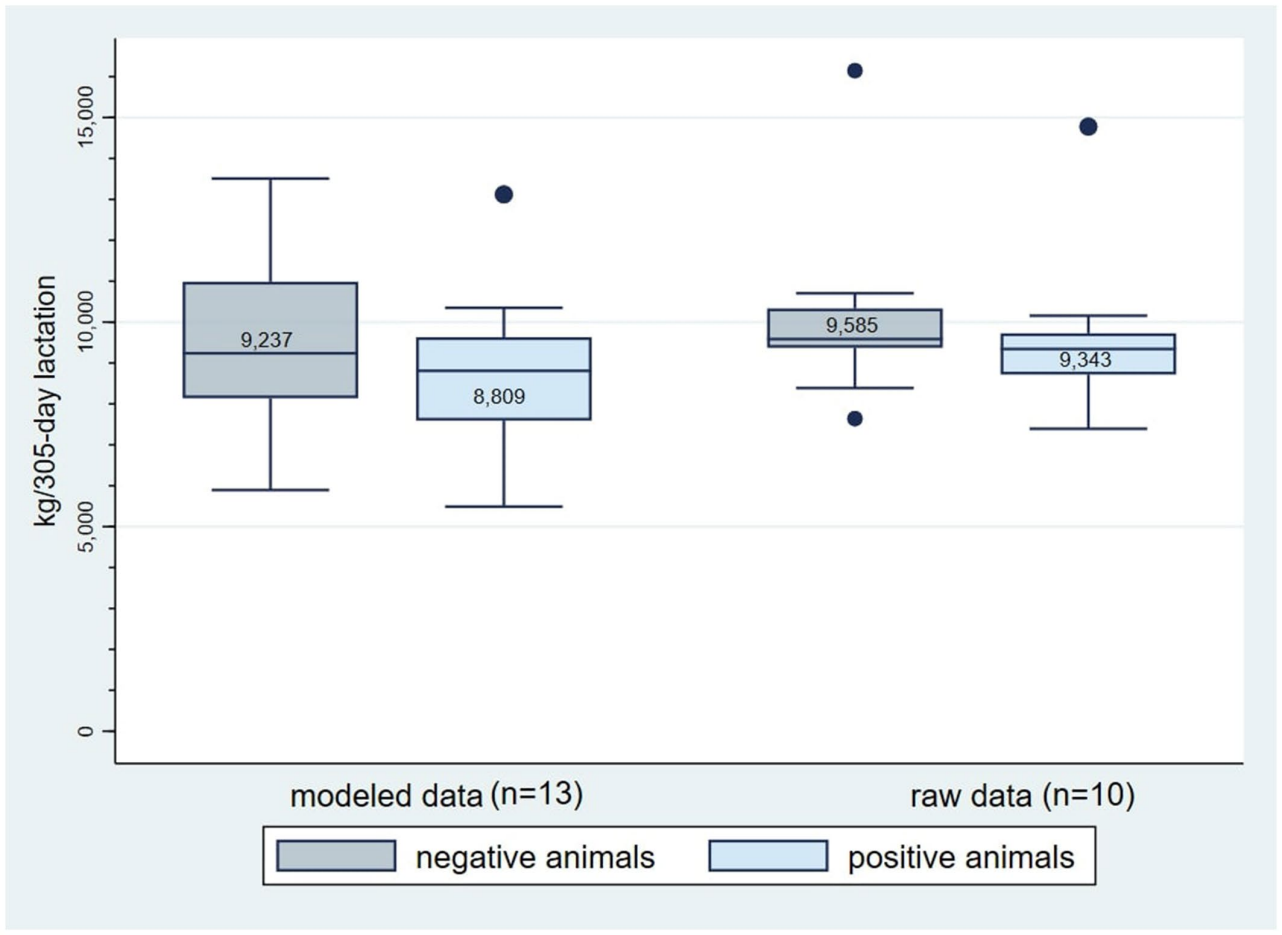
Average milk yield (kg/305-day lactation) of MAP negative and positive cows for modeled (*n* = 13) and raw (*n* = 10) data from publications included in the scoping review on associations between paratuberculosis and productivity in cattle. The number in the box indicates the median value.

The mean milk yield per positive or negative cow per 305-day lactation was reported in 13 and ten studies for modeled and raw data, respectively (Figure 4).

Expressed as percentages, the median milk yield decrease per lactation was −6.73% (95% confidence interval – CI −3.67, −11.46) for modeled and −4.29% (95%CI −1.78, −8.32) for raw data.

In the twelve studies in which the association between MAP infection and milk yield depending on parity was investigated (Supplementary Table S1), a median difference of −491.05 kg/lactation was found in parity one, −373.60 kg/lactation in parity two and −434.95 kg/lactation in parity three or higher (Table 7).

**Table 7:** Difference in milk yield (kg/305-day lactation) of paratuberculosis positive compared to paratuberculosis negative cows in parity 1, 2 and ≥ 3, based on 11 studies included in our scoping review on associations between paratuberculosis and productivity in cattle.

Parity	Number of publications	Minimum	Maximum	Median	95% confidence interval
Parity 1	12	−1,803	125.6	−491.05	−796.8559; −30.10905
Parity 2	12	−1,942.85	1,878.8	−373.6	−807.2795; −232.6945
Parity ≥3	12	−1,695.8	1,878.8	−434.95	−972.9231; 101.2425

### 3.2 Other milk parameters

Significantly higher SCC in MAP positive animals or herds than in negative animals or herds were reported in several studies (33, 42, 62, 66–68). Ozsvari et al. (42), for example, found that milk ELISA positive cows had on average a higher SCC by 41.4 × 1,000 cells/ml, which is equivalent to an increase of 35.8%. Sibley et al. (44) found similar results in their age-matched case control study, where cows with three subsequent positive milk ELISA tests had an average SCC of 238,000 cells/ml and the negative age cohort an average of 178,000 cells/ml. Significant differences in SCC between MAP positive and negative cows were not observed in other studies (27, 29, 32, 41, 46, 54).

The association between MAP infection and the amount of produced milk fat and protein were investigated in 16 publications (Table 8). Studies, in which effects were not quantified, (61, 62), where milk fat and protein were not looked at individually (65), where the positivity level was not dichotomous (54) and where the positivity level was defined on herd and not on individual animal level (64) are not listed in the table. Differences in milk protein of modeled data between PTB positive and negative animals at dichotomous level (Table 8) ranged from −27.94 kg/lactation to 1.82 kg/lactation with a median of −14.41 kg/lactation per positive cow (Table 9). Differences for milk fat ranged from −34.00 kg/lactation to 47.90 kg/lactation with a median of −13.13 kg/lactation per positive cow (Table 9). Pritchard et al. (67) explored the change of milk fat and protein in parity one to three. They found a significant decrease in milk fat of −1.4%, −4.0%, and −5.5% in cows at high risk for PTB (at least two adjacent milk ELISA tests MAP positive) and a decrease in milk protein of −1.3, −3.7%, and −4.9% in parity one, parity two and parity three, respectively.

**Table 8:** Data from studies (*n* = 11) included in our scoping review, in which associations between paratuberculosis and the amount of produced milk protein and milk fat were investigated dichotomously (positive vs. negative).

Test	Significance	Method	Country	Number of animals	Number of cases	Protein/fat quantification	Mean effect milk protein (kg/305-d lactation)	Mean effect milk fat (kg/305-d lactation)	Reference
Serum ELISA	Yes	Modeled	USA	4,375	295	kg/lactation	−8.31	−10	(27)
			Canada	689	130	kg/305-d lactation	−10	c	(32)
	No/not stated	Modeled	Canada	689	130	kg/305-d lactation	c	−11	(32)
			USA	1,653	147	%	−12.64^a,b^	−13.14^a,b^	(29)
			Israel	4,694	-	kg/305-d lactation	−9.8	−11.2	(34)
			USA	533	116	kg/305 ME	35.24	−18.54	(47)
		Raw	France	454	26	%	0.5^a,b^	7.79^a,b^	(31)
			France	424	48	%	−2.8 ^a,b^	−2.17 ^a,b^	(31)
			USA	1,653	147	%	−11.29^a,b^	−12.42 ^a,b^	(29)
Fecal culture	Yes		USA	4,375	115	kg/lactation	−18.03	−23.25	(27)
		Modeled	Canada	689	72	kg/305-d lactation	−22	−34	(32)
			Germany	9,367	1,136	kg/305-d lactation	−9^a^	−11^a^	(37)
	No/not stated	Modeled	USA	533	116	kg/305 ME	1.82	47.9	(47)
			Germany	4,627	1,382	%	−16.18^a,b^	−13.115^a,b^	(36)
Milk ELISA	Yes		Canada	689	77	kg/305-d lactation	−17	−18	(32)
		Modeled	Hungary	4,341	165	%	−27.94^b^	−24.8 ^b^	(42)
PCR (milk, serum, fecal)	Yes	Raw	Hungary	30	20	%	c	15.3^a,b^	(46)
	No/not stated	Raw	Hungary	30	20	%	−42.43^a,b^	c	(46)
Serum ELISA & fecal culture	Yes	Modeled	USA	4,375	345	kg/lactation	−9.49	−11.46	(27)
	No/not stated	Modeled	USA	533	116	kg/305 ME	45.53	24.57	(47)

^a^Calculated values based on given values (difference of healthy animals from that of the diseased animals). ^b^Unit converted to kg/305-day lactation. ^c^Study listed twice due to different significance level (either milk fat or protein significant and the other not). 305 ME = 305-day mature equivalent.

**Table 9:** Minimum, maximum, and median of dichotomous milk protein and milk fat mean difference (kg/305-day lactation) of the studies, in which associations between paratuberculosis and changes in milk protein and milk fat were investigated included in our scoping review.

Parameter	Number of publications	Minimum	Maximum	Median	95% confidence interval
Milk protein	8	−27.94	1.82	−14.41	−23.93; −5.49
Milk fat	8	−34.00	47.90	−13.13	−27.79; 8.14

Furthermore, fecal culture or PCR positive animals were found to have a lower content of lactose in the milk in two studies (36, 46).

### 3.3 Culling rate and longevity

Out of 14 studies in which associations between PTB and culling rate were investigated, an increased culling rate for MAP positive animals or herds was reported in 13. In the study of Mõtus et al. (69), a MAP positive status of the herds was not associated with higher culling rates, but the mean age of culled cows was lower, although it was not significant (−6.18 months; 95% CI −12.98, −0.63; *p* = 0.075). Likewise, in the study of Ozsvari et al. (42), seropositive cows were culled on average 160.5 days (95% CI 117.5, 303.5 days, *p* < 0.0001) equivalent to 5.28 months earlier, and in Vázquez et al. (70) 8.88 months earlier (corresponding to a −13.1% reduction of culling age) than negative cows. No difference in longevity was found by Lombard et al. (54). Publications, in which risk ratios for culling rates were reported can be found in the Supplementary Table S2. Associations between MAP positive herds or animals and higher culling rates were also found in Diéguez et al. (71), Rad et al. (58), Ott et al. (57), Smith et al. (72) as well as in Wilson (41), who additionally compared the cull rates between positive and negative animals within each lactation; a higher culling rate for MAP positive animals was found in every case. Arrazuría et al. (73) and Mato et al. (74) examined specific culling reasons for herds and cows positive for MAP by serum ELISA. They both found significantly higher culling rates due to lack of productivity (Odds Ratio – OR 2.46; 95% CI 1.41, 4.30; *p* = 0.002; Hazard Ratio – HR 2.55; *p* = 0.004), due to infertility (OR 1.24; 95% CI 1.01, 1.52; *p* = 0.039; HR 4.64; *p* = <0.001) and due to death/emergency slaughter (OR 3.49; 95% CI 2.7, 4.51; *p* = <0.001; HR 1.88; *p* = 0.045). While Arrazuria et al. (73) also found higher culling rates in MAP positive herds due to lameness (OR 2.45; 95% CI 1.55, 3.87; *p* = <0.001), there was no significant difference in Mato et al. (74). A higher culling rate due to mastitis was not observed in either of these studies.

### 3.4 Fertility parameters

Various parameters affecting fertility in MAP positive animals have been described, such as a prolonged calving interval, extended service period or higher number of inseminations per conception. Of the studies where a difference in calving interval was reported, the calving interval was prolonged by approximately 30 days (with a median of 31.9 days) for MAP positive cows (Supplementary Table S3). Rad et al. (58) also found a longer calving interval for infected cows in the parity one, two and three or higher. However, this association was not significant, and they attributed this observation to a significantly extended service period (period between date of calving and date of successful conception). A difference in calving interval between MAP positive and negative animals or herds was not found in other studies (37, 64, 66, 67, 75).

A parameter coherent with the calving interval is the service period, also known as number of days open. However, the results of the individual studies on this parameter are contradictory. While MAP positive cows were found to conceive later than negative animals in some studies, a shorter service period was observed for MAP positive cows in others (Supplementary Table S4).

It has been reported in various studies that a MAP positive cow needed on average significantly more inseminations per conception than a MAP negative cow (42, 46). In the study of Jurkovich et al. (46), MAP positive cows needed on average 2.8 inseminations and MAP negative animals 1.4 inseminations (*p* = 0.015) per pregnancy. In Ozsvari et al. (42), MAP positive cows had 3.47 services per conception on average and MAP negative animals only 3.05. This corresponds to an increase of 13.7% (*p* = 0.0192). No difference in the number of services per pregnancy per cow was found in other studies (38, 58, 67). A significantly higher pregnancy rate (the percentage of nonpregnant cows that become pregnant over a 21 day period) was found for infected cows (1.39%, *p* = 0.0395) in Gonda et al. (27), while no difference between groups was seen in any of the other studies on PTB and pregnancy rate (46, 76, 77). Higher non-return rates for MAP positive cows were found by Marcé et al. (78, 79), while in the study of Raizman et al. (51) fecal culture positive cows and heavy shedders were 2.8 (OR 95% CI 1.4, 5.7) times less likely to be inseminated again. Furthermore, an association between positive MAP status and abortion was observed in several studies (58, 77). Higher likeliness of calving difficulties was also described by Mato et al. (74) for MAP positive cows (OR 2.74; *p* < 0.001).

### 3.5 Weaning weight, carcass weight, and slaughter value

A possible association between PTB and meat quantity and quality was investigated in several studies. A reduction in carcass weight of −39.78 kg or −12.4% (*p* = < 0.0001) (70) and −58.45 kg (*p* = < 0.001) (80) for serum ELISA positive cows was reported. The slaughter weight for serum ELISA positive cows was reduced by up to −10% and up to −15% if they were also fecal culture positive, resulting in a reduction of slaughter value of −17% and −31%, respectively (81). Similarly, Benedictus et al. (65), described a reduction of −30% in slaughter value for cows in the clinical stage of PTB, but a normal slaughter value for non-clinically affected cows. In another study, losses were estimated at US$ 296/per clinical case (49). Mato et al. (80) found that seropositive cows were more likely to have poor carcass quality. They were 3.85 times more likely to have a poor carcass conformation score instead of fair and they had significantly lower fat cover scores.

A lower weaning weight was reported for calves born from a MAP positive dam compared to calves born from a negative dam (Supplementary Table S5).

### 3.6 Association with other diseases

The co-occurrence of other diseases in MAP positive animals is common (51). A significant association between MAP infection and the incidence of mastitis was observed in four out of six studies. Diéguez et al. (71) found a significant difference in the incidence of mastitis between highly positive farms (five or more seropositive cows with a mean herd size of 54.7 cows) and negative farms, while there was no difference between positive farms (herds with two to four seropositive cows) and negative farms. Pritchard et al. (67), who analyzed associations between lactation number and mastitis, found a significantly higher incidence of mastitis in high risk cows (at least two adjacent milk ELISA tests MAP positive) in the second and third lactation, compared to low risk cows (all milk ELISA tests negative or one test positive but last test negative). In the study of Rossi et al. (68) serum ELISA and fecal culture MAP-positive cows had a significantly higher first clinical mastitis risk per lactation than MAP negative cows (HR = 1.89; 95% CI 1.53, 2.33; *p* = <0.001). On the contrary, Wilson (41) found significantly lower non-clinical (*p* = < 0.01) and clinical (*p* = 0.05) mastitis rates in fecal culture positive cows compared to fecal negative cows.

MAP positive cows were reported to be lame on average 3 months earlier than negative animals and were 2.7 times (OR 2.7; 95% CI 1.2, 6.0; *p* = 0.017) more likely to become lame, even more if they had strongly positive results in milk ELISA (S/P ratio of 30% or above) (OR 3.6; 95% CI 1.1, 11.2; *p* = 0.029) (82).

Furthermore, animals with clinical JD were reported to be three times more likely to have pneumonia (OR 3; 95% CI 1.0, 6.0; *p* = 0.02) (83), heavy fecal shedders were 3.6 times (OR 3.6; 95% CI 1.2, 11.0; *p* = 0.03) more likely to develop abomasal displacement, and a higher risk of milk fever was reported for moderate fecal shedders (OR 4.8; 95% CI 1.1, 22.0) (51).

No association between positive status in fecal culture and metritis, retained placenta or ketosis was observed (38, 83).

## 4 Discussion

The aim of our study was to provide a comprehensive overview of associations between MAP infection and production parameters in cattle. We identified 67 studies published between 1987 and 2021 reporting associations between PTB and 34 different production parameters. We found extensive research on the association between MAP infection and changes in milk production and milk composition. However, studies on the association of PTB and changes in meat production (e.g., slaughter weight, weaning weight, meat quality) and fertility (e.g., calving interval, service period number of insemination, abortion rate, non-return rate) are relatively scarce. Most of the studies are from North America (*n* = 33) and North-Western Europe (*n* = 25), while only very few studies could be included from Eastern Europe (*n* = 4), Asia (*n* = 4), Oceania (*n* = 1) and none from Central and South America and Africa. Studies from these regions would be crucial for an analysis of the economic impact of PTB in these countries, as the impact may vary due to different forms of production, breeds or husbandry. For example, most of the studies included in this work were conducted on high producing Holsteins, thus these results may not be extrapolated to other breeds and production systems. However, the lack of studies from these regions might also be due to a language bias, as only publications written either in English, French, German, or Slovenian were considered.

Most MAP infections remain subclinical for years and, therefore, losses in production are difficult to estimate. Nevertheless, many studies report that the chronic disease leads to a substantial loss in milk yield. Results of different studies show a median loss of −405 kg or a decrease of −6.73% for modeled data and −452 kg or a decrease of −4.29% for raw data per 305-day lactation per positive cow as assessed with various tests (while considering the result of fecal culture if available in case multiple tests were conducted in the same study). However, the extent of production losses varies as well depending on the sensitivity and specificity of the test(s) used and on the infection status of the tested animals. The most common tests used in research are serum ELISA and fecal culture, whereby specificity and sensitivity vary within the test categories, e.g., for different ELISA tests or fecal culture protocols (84). Results of the studies analyzed in our review show a median decrease of −279.63 kg/305-day lactation for serum ELISA-positive animals and a −583.35 kg/305-day lactation decrease for fecal culture positive animals. McAloon et al. (13) reported similar findings. In their systematic review and meta-analysis of the effect of PTB on milk yield, a decrease of −576.45 kg/305-day lactation for fecal culture or PCR positive cows and a decrease of −1.03 kg/day (equivalent to −314.05 kg/305-day lactation) per ELISA positive cow was calculated. These authors explained the lower effect for serum ELISA positive animals in comparison to fecal culture positive animals by the lower specificity of the serum ELISA test, which results in more false positive animals and, therefore, in an underestimation of the effect. Another factor affecting the extent of impact is the state of infection. Rieger et al. (10) calculated in their meta-analysis based on twelve studies a daily loss of −0.71 kg for serum ELISA positive animals (equivalent to −216 kg/305-day lactation). Smith et al. (30, 50) found that subclinically infected cows produced more milk than negative animals, while heavy shedding and strongly positive animals (>50 cfu in at least one tube) had the largest decrease in milk production (Table 5). Fewer days in milk were also reported for PTB positive cows (33, 38, 51), which would also have an impact on the milk production per lactation. It was not clear in all publications whether the positive and negative animals were from the same herds or different herds. This certainly also has an impact on the extent of the losses.

The effect of PTB on milk protein and milk fat is controversial. A decrease in milk fat and protein in positive animals, was observed in some studies, while an increase or no difference at all were seen in others. However, a negative association (decrease in milk protein) was reported in all studies in which a significant association between positive animals and milk protein was found. Overall, a median decrease of −14.41 kg protein/305-day lactation/positive cow was calculated for modeled data. Similarly, a significant decrease in milk fat was reported in most studies. Overall, a median decrease of −13.13 kg/305-day lactation/positive cow was calculated for modeled data. However, all these studies, which found a significant decrease in milk fat or protein, analyzed the total amount produced per lactation. This decrease might, therefore, also be due to lower milk production. PTB, as other chronic diseases, may lead to a weakened immune system (67). This may explain the susceptibility of infected animals to other diseases, such as mastitis, lameness or pneumonia, as it was observed in several studies. The higher rate of mastitis and poorer udder health may explain the higher SCC in MAP positive cows.

The chronic granulomatous gastroenteritis caused by MAP, causes a malabsorption syndrome, leading to malnutrition and muscle atrophy (85). Consequently, it may lead to a decrease in slaughter value and slaughter weight up to −31% for clinically infected cows. Furthermore, a decreased weaning weight between −2.3 (75) and up to −33.6 (86) kg/calf born to a positive dam was reported.

Whether MAP infection also has an influence on fertility is controversial. However, a prolonged calving interval by around 30 days, a longer service period, more inseminations per pregnancy or higher abortion rates for positive animals were observed in several studies. Rieger et al. (10) attributed the highest losses to a prolonged service period. In their meta-analysis, which was based on three studies, serum ELISA-positive animals had a longer service period of 14.93 days.

The production losses mentioned above may ultimately explain the earlier culling of around six months for positive animals and the fact that positive cows are 1.5 up to 3 times more likely to be culled per lactation.

Furthermore, the direct losses described above lead to further indirect costs, which also need to be taken into account for an economic impact assessment of the disease. Higher culling rates lead to higher costs for herd replacement, susceptibility to other diseases to higher veterinary costs, and higher SCC and poor carcass quality may lead to lower rewards.

This scoping review has some limitations. Studies were reviewed for the relevance for our research question, but their quality was not fully assessed.

Furthermore, no meta-analysis was performed for different reasons. First, there is a large diversity between studies, as for example in study design, methodology, case definition or applied diagnostic tests. Second, for most parameters, there was not enough data to perform a meta-analysis. Sufficient data would have been available for milk yield, where a meta-analysis has been published recently (13). However, study results were standardized and summarized as far as possible, and a comprehensive overview of the existing literature was provided. The median and range of some production parameters were assessed for raw and modeled data separately. A negative association of PTB on production parameters was observed in most studies. However, a publication bias for opposite results cannot be ruled out. In addition, the negative association may also be the other way around. Reproductive failure, lower production and higher probability of other diseases are also indicators of poor management practices (87–89). Poor husbandry practices, may lead to stress, malnutrition, and an immunocompromised state in animals, and may exacerbate the severity of the course of MAP infections. Poor conditions could favor the spread of MAP within a herd through fecal-oral transmission. Therefore, animals from farms with poor husbandry may be more susceptible to infection after exposition to and ingestion of MAP, leading to increased shedding and seropositivity. In addition, herds with inadequate biosecurity measures may be more exposed to the entry of infected animals or contaminated materials, leading to a higher prevalence of paratuberculosis.

MAP-infected animals can show various manifestations of infection which are influenced by the progression and stage of the disease, but also other factors as for example genetic predisposition to the pathogen, age at the time of infection and any prior exposure to other environmental mycobacteria (90). All these factors certainly affect the extent of production losses and could not be analyzed in detail in the scope of this review.

## 5 Conclusion

Sixty-seven existing studies on the association of PTB on productivity in cattle from 17 different countries were identified and analyzed. This scoping review confirms that PTB is negatively associated with various production parameters and is of great relevance for productivity in cattle. This needs to be taken into account when estimating the economic burden of MAP infection. The review has revealed that associations vary depending on disease status (clinical vs. non-clinical, MAP shedding vs. latent infection), which makes an impact assessment more complex. Not only disease status but also the many different diagnostic tests and their variability in sensitivity and specificity are a challenge. More studies on the association between PTB and productivity would be beneficial, especially regarding fertility and meat production, cow breeds other than Holsteins, and better insights into the associations of changes in productivity and the different infection status (e.g., early or latent forms of infection).

## Author contributions

SG: Data curation, Formal analysis, Methodology, Writing – original draft, Writing – review & editing, Investigation, Visualization. TK: Data curation, Formal analysis, Methodology, Writing – review & editing, Conceptualization, Funding acquisition, Investigation. AB: Formal analysis, Writing – review & editing. LC: Conceptualization, Writing – review & editing, Funding acquisition, Methodology. GS-R: Writing – review & editing, Conceptualization, Funding acquisition, Methodology, Project administration, Supervision. MM: Funding acquisition, Writing – review & editing, Conceptualization, Methodology. MO: Conceptualization, Writing – review & editing, Funding acquisition, Methodology. BT: Conceptualization, Formal analysis, Supervision, Writing – review & editing, Investigation, Methodology, Project administration.
