# Supplementary material for: A scoping review on associations between paratuberculosis and productivity in cattle
Source: Front Vet Sci. 2024 May 2;11:1352623. doi: 10.3389/fvets.2024.1352623 (PMC11097669; doi:10.3389/fvets.2024.1352623)
Supplement: Supplementary file 1 [file Table_1.docx]

Supplementary material: A scoping review on associations between paratuberculosis and productivity in cattle

Silja Griss^1,2*^ Tanja Knific^3^, Anne Buzzell^1^, Luís Pedro Carmo^4^, Gertraud Schüpbach-Regula^1^, Mireille Meylan^5^, Matjaž Ocepek^6^, Beat Thomann^1^

^1^Veterinary Public Health Institute, Vetsuisse Faculty, University of Bern, Bern, Switzerland

^2^Graduate School for Cellular and Biomedical Sciences, University of Bern, Bern, Switzerland

^3^Veterinary Faculty, Institute of Food Safety, Feed and Environment, University of Ljubljana, Ljubljana, Slovenia

^4^Norwegian Veterinary Institute, Ås, Norway

^5^Clinic for Ruminants, Vetsuisse Faculty, University of Bern, Bern, Switzerland

^6^Veterinary Faculty, Institute of Microbiology and Parasitology, University of Ljubljana, Ljubljana, Slovenia

Table S1: Data from studies (n=11) which investigated the association between paratuberculosis and changes in milk production for different parities (kg/305-day lactation) included in our scoping review

| **Test** | **Milk quantification** | **Parity 1 (p-value)** | **Parity 2 (p-value)** | **Parity ≥ 3 (p-value)** | **Reference** |
| --- | --- | --- | --- | --- | --- |
| Milk ELISA | kg/day | -1,128.5^b^ (<0.001) | -823.5^b^ (0.007) | -244^b^ (0.46) | (Kudahl, Nielsen and Sørensen, 2004) |
|  | kg/305-d lactation | -530.7^b^ (0.0505) | -1,942.85^b^ (<0.0001) | -1,695.8^b^ (<0.0001) | (Ozsvari *et al.,* 2020) |
|  | kg/305-d lactation | -253.9 (0.0138) | -411.7 (<0.0001) | -381.9 (0.0008) | (Sorge *et al.,* 2011) |
|  | kg/day | -103.7^b^ (<0.05) | -335.5^b^ (<0.05) | -488^b^ (<0.05) | (Pritchard *et al.,* 2017) |
| Serum ELISA | kg/305-d lactation | 125.6 (0.004) | 45.8 (0.004) | 135.8 (0.004) | (Tiwari *et al.,* 2007) |
|  | kg/test-day | -481.9^b^ (<001) | -671^b^ (<0.001) | -1,006.5^b^ (<0.001) | (Beaudeau *et al.,* 2007) |
|  | kg/305-d lactation | -573 (<0.05) | -226 (>0.05) | 1,476 (<0.05) | (VanLeeuwen, Keefe and Tiwari, 2002) |
|  | kg/day of life | -500.2^b^ (<0.0001) | -298.9^b^ (0.139) | -189.1^b^ (0.546) | (Villarino, Scott and Jordan, 2011) |
|  | kg/day | -21.35^a,b^ (>0.05) | -442.25^a,b^ (>0.05) | 1,878.8^a,b^ (>0.05) | (Wiszniewska-Łaszczych *et al.,* 2020) |
|  | kg/day | -823.5^a,b^ (0.008) | 1,878.8^a,b^ (>0.05) | -622.2^a,b^ (>0.05) | (Wiszniewska-Łaszczych *et al.,* 2020) |
| Fecal culture | kg/305-d lactation | -1,803 (<0.05) | -1,193 | -1,105 | (Raizman, Fetrow and Wells, 2009) |
|  | lbs/305 ME | 112.04^a,b^ (>0.05) | -288.94^a,b^ (<0.05) | -690.82^a,b^ (<0.05) | (Wilson, 1995) |

a) Calculated values based on given value difference of healthy animals from that of the diseased animals)

b) Unit converted to kg/305-day lactation

305 ME = 305-day mature equivalent

Table S2: Culling risk for any reason for MAP positive animals compared to MAP negative animals found in the studies (n=4) included in our scoping review on the associations between paratuberculosis and productivity in cattle

| **Test** | **Number of animals** | **Number of cases** | **Type of ratio** | **Culling risk (95% confidence interval)** | **P-value** | **Reference** |
| --- | --- | --- | --- | --- | --- | --- |
| Serum ELISA | 1,556 | 241 | Relative risk | 1.58 (1.32,1.89) | <0.05 | (Goodell *et al.*, 2000) |
|  | 689 | 130 | Hazard ratio | 1.7 (1.4,2.3) | 0.033 | (Hendrick *et al*., 2005) |
|  | 3,531 | NA | Hazard ratio | 1.38 (1.05,1.81) | 0.019 | (Tiwari *et al*., 2005) |
| Milk ELISA | 689 | 77 | Hazard ratio | 2.3 (1.8,3.0) | 0.001 | (Hendrick *et al*., 2005) |
| Fecal culture | 689 | 72 | Hazard ratio | 3.2 (2.5,4.2) | <0.001 | (Hendrick *et al*., 2005) |
|  | 1,048 | 84 | Odds ratio | 3 (1.6,5.8) | - | (Raizman, Fetrow and Wells, 2009) |

Table S3: Comparison of calving interval between MAP positive and negative animals found in the studies (n=4) included in our scoping review on the associations between paratuberculosis and productivity in cattle

| **Test** | **Calving interval MAP positive animals (days)** | **Calving interval MAP negative animals (days)** | **Mean difference (days)** | **P-value** | **Reference** |
| --- | --- | --- | --- | --- | --- |
| Milk PCR | 458.4 | 428.9 | +29.5 | 0.057 | (Ansari-Lari, Haghkhah and Mahmoodi, 2012) |
| Fecal PCR | 437 | 365 | +72a | 0.006 | (Jurkovich *et al.*, 2016) |
| Milk ELISA | 470.9 | 437.1 | +33.8 | 0.0013 | (Ozsvari *et al*., 2020) |
| Milk ELISA | 416 | 386 | +30 a | - | (Sibley, Orphin and Pearse, 2012) |

a) mean difference calculated

Table S4: Comparison of service period between MAP positive and negative animals found in the studies (n=6) included in our scoping review on the associations between paratuberculosis and productivity in cattle

| **Case definition** | **Service period MAP positive animals (days)** | **Service period MAP negative animals (days)** | **Mean difference** | **P-value** | **Reference** |
| --- | --- | --- | --- | --- | --- |
| Serum ELISA positive (dichotomous) | - | - | +28 | 0.02 | (Johnson-Ifearulundu *et al.,* 2000) |
| Fecal PCR positive | 168.9 | 84.6 | +84.3^a^ | 0.003 | (Jurkovich *et al*., 2016) |
| Milk ELISA positive | 164.6 | 141.3 | +23.2 | 0.0012 | (Ozsvari *et al*., 2020) |
| Fecal culture positive | 122 | 149 | -27^a^ | non-significant | (Raizman *et al*., 2007a) |
| Fecal culture positive | 118.9 | 151.6 | -32.4^a^ | non-significant | (Raizman, Fetrow and Wells, 2009) |
| Serum ELISA inconclusive | 185 | 196 | -11^a^ | non-significant | (Lombard *et al*., 2005b) |
| Serum ELISA positive | 206 | 196 | 10^a^ | non-significant | (Lombard *et al.,* 2005b) |
| Serum ELISA strong positive | 161 | 196 | -35^a^ | significant | (Lombard *et al*., 2005b) |

Table S5: Difference of adjusted 205-day weaning weight (kg) between a calf of an MAP positive dam and a calf of a MAP negative dam found in the studies (n=3) included in our scoping review on the associations between paratuberculosis and productivity in cattle

| **Test** | **Difference in weaning weight per calf (kg of adjusted weaning weight/ 205 days)** | **P-value** | **Reference** |
| --- | --- | --- | --- |
| Serum ELISA | -5.53 | 0.016 | (Bhattarai *et al.,* 2013) |
|  | -2.3 | 0.01 | (Elzo *et al*., 2009) |
| Fecal culture | -33.6 | <0.001 | (Bhattarai *et al*., 2013) |
